# Usefulness of Nutritional Assessment Indicators in Predicting Treatment Discontinuation Due to Adverse Events from PARP Inhibitors in Ovarian Cancer Patients

**DOI:** 10.3390/cancers16213602

**Published:** 2024-10-25

**Authors:** Yoshiaki Tanaka, Daisuke Inoue, Hideaki Tsuyoshi, Yuriko Nakamura, Masato Kato, Masataka Kato, Kentaro Niwa, Kenji Yashiro, Makoto Orisaka, Yoshio Yoshida

**Affiliations:** 1 Department of Obstetrics and Gynecology, Faculty of Medical Sciences, University of Fukui, 23-3 Matsuokashimoaiduki, Yoshida-Gun, Eiheiji-Cho, Fukui 910-1104, Japan; yoky@u-fukui.ac.jp (Y.T.); ddd@u-fukui.ac.jp (D.I.); gth@u-fukui.ac.jp (H.T.); orisaka@u-fukui.ac.jp (M.O.); 2 Department of Obstetrics and Gynecology, Fukui Prefectural Hospital, 2-8-1 Yotsui, Fukui 910-0846, Japan; 3 Department of Obstetrics and Gynecology, Fukui-Ken Saiseikai Hospital, Fukui 918-8503, Japan; 4 Department of Obstetrics and Gynecology, Fukui Red Cross Hospital, 2-4-1 Tsukimi, Fukui 918-8011, Japan; 5 Department of Obstetrics and Gynecology, Central Japan International Medical Center, 1-1 Kenkonomachi, Minokamo 505-8510, Japan; 6 Department of Obstetrics and Gynecology, Ishikawa Prefectural Central Hospital, 2-1 Kuratsuki Higashi, Kanazawa-City 920-8530, Japan

**Keywords:** ovarian cancer, PARP inhibitor, Prognostic Nutritional Index, Controlling Nutritional Status score, modified Glasgow Prognostic Score, treatment discontinuation

## Abstract

Background: Nutritional status is an important factor influencing toxicity of treatment. Nutritional assessment indicators such as the Prognostic Nutritional Index (PNI), Controlling Nutritional Status (CONUT) score and modified Glasgow Prognostic Score (mGPS) have been reported to be associated with treatment-related adverse events (AEs) for various malignancies. However, there are no reports investigating the relationship between nutritional status and AEs from poly-(ADP-ribose) polymerase (PARP) inhibitors (PARPi), which are widely used in recent years as maintenance therapy for ovarian cancer. Objective: The primary objective was to investigate the usefulness of nutritional assessment indicators in predicting treatment discontinuation due to AEs from PARPi. Methods: This multicenter retrospective study included patients diagnosed with ovarian cancer who received maintenance therapy with PARPi from January 2018 to December 2023. PNI, CONUT score, and mGPS were calculated based on hematological parameters measured within 7 days before the start of PARPi therapy. Results: A total of 272 patients received maintenance therapy with PARPi during the period, but due to the absence of the blood collection of albumin levels within one week or other exclusion criteria, 71 patients were finally included in this analysis. AEs were seen in 59 patients (83.1%), including 25 (35.2%) severe events (grade ≥3 in Common Terminology Criteria for Adverse Events v5.0). Eighteen patients (25.4%) discontinued treatment due to PARPi-related AEs. Low PNI (<48.44) and high mGPS (≥1) were predictors of treatment discontinuation in both univariate and multivariate analyses. CONUT was not a significant predictor in this study. Conclusions: Our study suggested that PNI and mGPS can predict the risk of treatment discontinuation due to PARPi-related AEs before starting maintenance therapy. This insight opens avenues for more personalized treatment plans, potentially improving patient outcomes.

## Simple Summary

Nutritional assessment indicators have been recognized as useful in predicting treatment-related adverse events. In maintenance therapy with PARP inhibitors in ovarian cancer, treatment discontinuation due to treatment-related adverse events has been reported, but factors predicting these events are not identified. Our study suggested that PNI and mGPS can predict the risk of treatment discontinuation due to PARP inhibitor-related adverse events before starting maintenance therapy. This insight opens avenues for more personalized treatment plans, potentially improving patient outcomes.

## 1. Introduction

Ovarian cancer remains a significant cause of gynecological cancer-related deaths worldwide, which are particularly increasing in Japan [1]. Epithelial carcinoma, particularly high-grade serous carcinoma, constitutes around 95% of cases. In addition, more than 75% of these cases are diagnosed at an advanced stage [2]. Despite initial responsiveness to platinum-based chemotherapy combined with taxanes, the relapse rate for advanced-stage disease exceeds 80% [3]. With each additional treatment, progression-free survival (PFS) decreases, while the cumulative risk of adverse events (AEs) increases with each new round of chemotherapy, and many patients die within 5 years of diagnosis [4,5]. This situation underscores the need for effective maintenance therapy.

Maintenance therapy offers the opportunity to prolong remission and the chemotherapy-free interval and helps delay toxic effects associated with the next cycle of chemotherapy. Two clinical trials of maintenance therapy for recurrent ovarian cancer have been reported recently. The first was a clinical trial using olaparib, a potent oral poly-(ADP-ribose) polymerase (PARP) inhibitor (PARPi). That study, the phase 3 SOLO 2 trial, showed that olaparib significantly prolonged PFS in patients with platinum-sensitive recurrent ovarian cancer with germline BRCA1/2 mutations who responded to platinum-based chemotherapy [6]. The second clinical trial involved niraparib, a highly selective PARP 1 or 2 inhibitor. The efficacy of niraparib as maintenance treatment for patients with relapsed ovarian cancer who had experienced complete or partial response to platinum-based chemotherapy was evaluated in the phase III ENGOT-OV16/NOVA trial [7]. The results showed that niraparib treatment significantly prolonged PFS compared with the placebo, irrespective of germline BRCA or homologous recombination loss status. However, a high incidence of AEs has also been reported, with the rate of Grade (G)3 or worse AEs ranging from approximately 40% for olaparib to more than 70% for niraparib. These high incidences of AEs are mainly attributable to hematological toxicities such as anemia, neutropenia, and thrombocytopenia [6,7]. The appearance of AEs often necessitates an interruption or reduction in treatment, with more than 10% of patients forced to discontinue treatment. However, no biomarkers to predict treatment discontinuation due to PARPi-related AEs have yet been described.

Various predictors of treatment-related AEs have been investigated for conventional chemotherapy. In particular, associations with nutritional status have often been reported. Nutritional assessment indicators such as the Prognostic Nutritional Index (PNI), Controlling Nutritional Status (CONUT) score and modified Glasgow Prognostic Score (mGPS), all of which mainly comprise a score for the serum albumin level, are tools to objectively evaluate nutritional status, and these indicators have been reported to be associated with treatment-related AEs [8,9,10]. Relationships between these nutritional indicators and chemotherapy-induced AEs have also been reported [11,12]. PNI has recently been reported as a useful value for predicting AEs caused by immune checkpoint inhibitors [13,14].

Given the critical impact of nutritional status on cancer treatment outcomes and the existing void in predictive tools for PARPi-induced AEs, our study investigated the utility of the PNI, CONUT score and mGPS nutritional assessment indicators as predictors for treatment discontinuation due to AEs in PARPi therapy for ovarian cancer. This approach was aimed at personalizing and enhancing patient care by identifying individuals at heightened risk for AEs, thereby informing more tailored treatment decisions.

## 2. Materials and Methods

### 2.1. Participants and Study Design

This multicenter retrospective study focused on patients who received PARPi (olaparib or niraparib) maintenance therapy for ovarian cancer at University of Fukui Hospital, Fukui Prefectural Hospital, Ishikawa Prefectural Central Hospital, Central Japan International Medical Center, Fukui Red Cross Hospital, and Fukui-ken Saiseikai Hospital) from January 2018 to December 2023. Adhering to the STROBE checklist for cohort studies, this research aimed to evaluate the predictive value of nutritional assessment indicators for treatment discontinuation due to AEs from PARPi. Eligibility for PARPi administration was limited to patients who had an achieved partial response (PR) or complete response (CR) according to the Response Evaluation Criteria in Solid Tumors (RECIST 1.1) [15] following initial treatment with platinum-based chemotherapy. The following exclusion criteria were applied: patients who have been previously prescribed PARPi; patients with other cancers; patients with other complications that may cause hypoalbuminemia, such as renal disease that causes nephrotic syndrome, protein-leaking gastrointestinal disease, or liver disease; patients who have no data on serum albumin levels within one week prior to starting PARPi; and patients who are interrupted in follow-up due to transfer to a different hospital or other reasons.

### 2.2. Nutritional Assessment Indicators

Nutritional status was assessed using PNI, CONUT score and mGPS, calculated based on hematological parameters measured within 1 week before starting PARPi. Hematological parameters were collected from electronic medical records.

The PNI is based on total lymphocyte and serum albumin, calculated as follows: 10 × albumin (g/dL) + 0.005 × total lymphocyte count (/μL). Lower scores indicate poorer nutritional status [16].

The CONUT score is calculated by scoring serum albumin, total cholesterol, and total lymphocyte count, and summing the results. These scores are calculated as follows: (albumin > 3.5 g/dL) = 0; (3.0 g/dL < albumin < 3.5 g/dL) = 2; (2.5 g/dL < albumin < 3.0 g/dL) = 4; (albumin < 2.5 g/dL) = 6; (total cholesterol > 180 mg/dL) = 0; (140 mg/dL < total cholesterol < 180 mg/dL) = 1; (100 mg/dL < total cholesterol < 139 mg/dL) = 2; (total cholesterol < 100 mg/dL) = 3; (total lymphocyte count > 1600/mL) = 0; (1200/mL < total lymphocyte count < 1600/mL) = 1; (800/mL < total lymphocyte count < 1200/mL) = 2; and (total lymphocyte count < 800/mL) = 3. Based on the total score, the nutritional status of the patient is categorized as normal (total score 0–1), light undernutrition (total score 2–4), moderate undernutrition (total score 5–8), or severe undernutrition (total score 9–12) [17].

The mGPS is based on C-reactive protein (CRP) and serum albumin, scored at three levels from 0 to 2. The score is calculated as follows: (CRP < 0.5 mg/dL and albumin > 3.5 mg/dL) = 0; (CRP > 0.5 mg/dL or albumin < 3.5 mg/dL) = 1; and (CRP > 0.5 mg/dL and albumin < 3.5 mg/dL) = 2. Higher scores indicate poorer nutritional status [18].

### 2.3. Adverse Events

Hematological toxicities were evaluated from hematological parameters based on the Common Terminology Criteria for Adverse Events (CTCAE) v5.0, and severe events were defined ≥grade 3 adverse events [19]. Other AEs were confirmed from medical record entries made by the attending physicians. For all patients, all records were checked from the initiation of PARPi administration to the end of either maintenance therapy or follow-up.

### 2.4. Statistical Analyses

**Figure 1:**
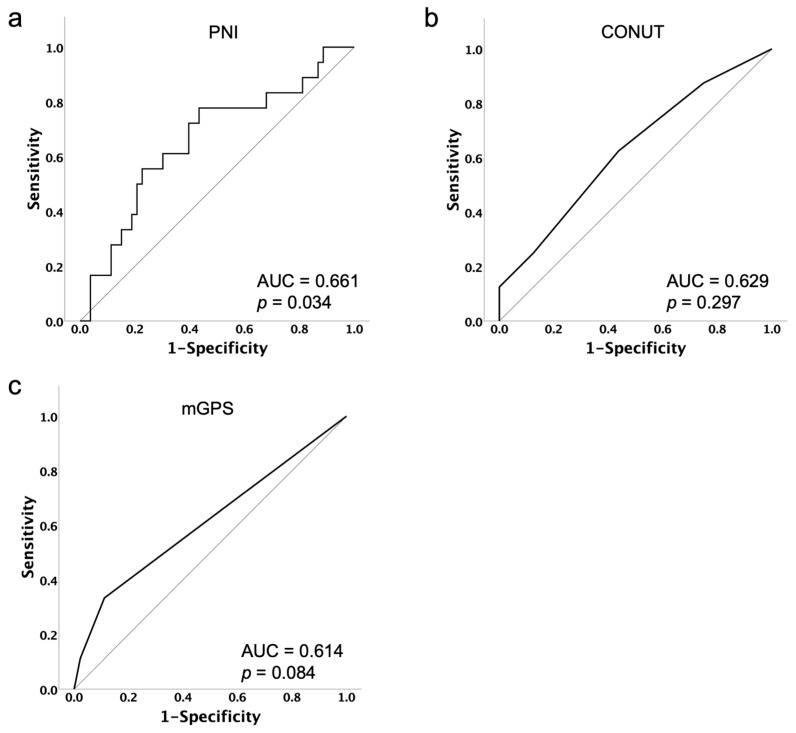
Receiver operating characteristic curves of PNI (**a**), CONUT (**b**) and mGPS (**c**) with respect to the prediction of treatment discontinuation due to PARPi-related AEs. The optimal cut-off was identified using the Youden Index from the ROC analysis as 48.44 for PNI, 2 for CONUT and 1 for mGPS.

PNI, CONUT, mGPS, age, Body Mass Index (BMI), International Federation of Gynecology and Obstetrics (FIGO) stage, previous lines of platinum therapy, differences in initial dose, and serum albumin levels before PARPi administration were analyzed. For each nutritional assessment indicator, the optimal cut-off value for predicting treatment discontinuation due to PARPi-related AEs was determined by the Youden Index of the receiver operating characteristic (ROC) curve (Figure 1).

According to the optimum cut-off values, patients were stratified as low- and high-PNI (≤48.44 and >48.44), low- and high-CONUT (<2 and ≥2), or low- and high-mGPS (<1 and ≥1) groups. Parameters other than the nutritional assessment indicators were stratified by referring to previous reports [20,21]. The time from the start of maintenance therapy until treatment was discontinued due to PARPi-related AEs was defined as time to treatment discontinuation (TTD). TTD curves were calculated using the Kaplan–Meier method, and the log-rank test was employed to assess differences. Cox regression models were applied to find independent indicators associated with TTD. Multivariate analyses were performed for each nutritional assessment indicator that was significant in the univariate analysis, adding age, BMI and previous lines of platinum therapy. Multicollinearity was assessed using the Variance Inflation Factor (VIF) for each nutritional assessment indicator. IBM SPSS Statistics version 28 was used for all statistical analyses. A *p* value < 0.05 was considered statistically significant.

### 2.5. Ethical Consideration

This retrospective study, based on a review of existing medical records, adhered to the principles of the Helsinki Declaration and was approved by the Ethics Review Committee of University of Fukui (approval no. 20240041).

## 3. Results

### 3.1. Study Population and Patient Characteristics

**Figure 2:**
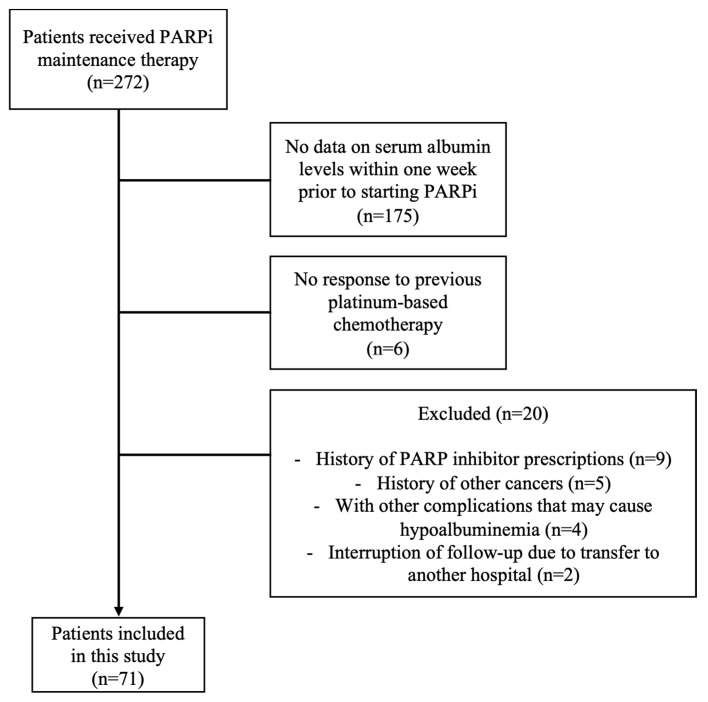
Patient flow diagram for analysis.

A total of 272 patients received maintenance therapy with PARPi during the period, but due to the absence of the blood collection of albumin levels within one week or other exclusion criteria, 71 patients were finally included in this analysis (Figure 2).

The median age was 63 (interquartile range: 56 to 69.5), and 41 patients (57.7%) were older than 65. The median BMI was 21.91 kg/m^2^ (interquartile range: 19.6 to 24.6 kg/m^2^), and 12 patients (16.9%) had a BMI below 18.5 kg/m^2^. A total of 66 patients had FIGO stage III or higher at the time of initial diagnosis, and 19 (26.8%) patients had received three or more lines of platinum therapy. Olaparib was administered to 53 patients (74.6%) and niraparib to 18 patients (25.4%). In 5 (7.0%) patients, physicians reduced the initial dose of PARPi due to bone marrow suppression attributed to preceding treatment or age (Table 1).

### 3.2. Details of AEs and Cases of Discontinuation Due to AEs Caused by PARPi

The occurrence rate AEs of all grades was 83.1%, with severe events (≥G3) constituting 35.2% (Table 2).

A total of 18 of the 71 patients (25.4%) required treatment discontinuation due to PARPi-related AEs. Anemia was the most common reason, with discontinuation in seven patients. The average time to treatment discontinuation was 152.1 days, with the shortest 15 days and the longest 889 days. A total of 15 (83%) patients discontinued maintenance therapy within six months and 17 (94%) within one year (Table 3).

### 3.3. TTD

**Figure 3:**
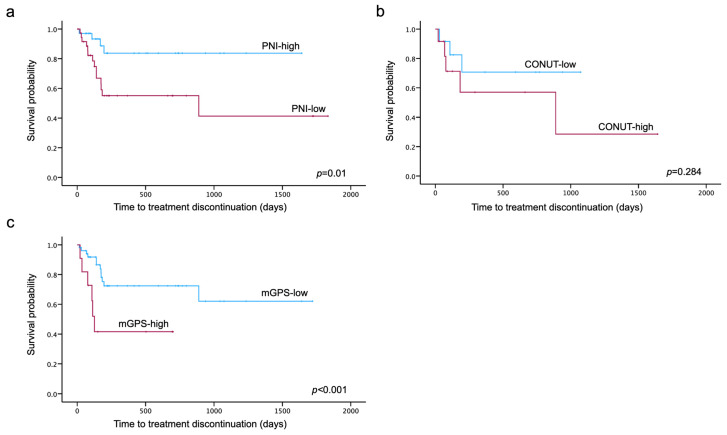
Kaplan–Meier curves for PNI (**a**), CONUT (**b**) and mGPS (**c**) until treatment discontinuation due to PARPi-related AEs. Divided into two groups according to the cut-off values obtained by the ROC analysis, Kaplan–Meier curves were generated for each nutritional assessment indicator. The log-rank test showed that time to treatment discontinuation was significantly shorter in the low-PNI and high-mGPS groups (*p* = 0.01, *p* < 0.001).

Kaplan–Meier curves for TTD were generated for each nutritional assessment indicator. The log-rank test showed that TTD was significantly shorter in the low-PNI and high-mGPS groups (*p* = 0.01, *p* < 0.001) (Figure 3).

### 3.4. Predictors for Treatment Discontinuation Due to AEs from PARPi

In addition to nutritional assessment indicators, potential predictors like age, BMI, FIGO stage, previous lines of platinum therapy and differences in initial dose before PARPi administration were analyzed using Cox regression analysis to identify predictors of treatment discontinuation. Low PNI and high mGPS emerged as significant risk factors in the univariate analysis (*p* = 0.017 and *p* = 0.013, respectively), while CONUT did not show a significant association. Low PNI and high mGPS remained significant risk factors in the multivariate analysis, along with age, BMI and previous lines of platinum therapy (Table 4). To assess for multicollinearity, the VIF was calculated for each nutritional assessment indicator (PNI: 1.93; mGPS: 1.11; and CONUT: 2.37), indicating no significant multicollinearity among the nutritional indicators.

Subgroup analyses were conducted for the 40 patients who received maintenance therapy with olaparib alone, revealing high mGPS as a risk factor in univariate and multivariate analysis (Table 5).

## 4. Discussion

Our study uniquely reveals that nutritional assessment indicators, notably PNI and mGPS, prior to the start of maintenance therapy were predictors of treatment discontinuation due to PARPi-related AEs in patients with ovarian cancer receiving maintenance therapy with PARPi. These results suggest that before starting PARPi maintenance therapy, nutritional status should be assessed, with closer monitoring of those patients showing low PNI (<48.44) or high mGPS (≥1). This finding contributes to a growing body of evidence underscoring the impact of nutritional status on treatment outcomes in oncology, particularly the utility of nutritional assessment indicators in forecasting treatment-related toxicities and survival rates across various cancers [11,22,23].

Several studies have previously identified low PNI as a predictor of adverse outcomes across different treatment modalities and cancer types. Huang et al. highlighted the role of PNI in predicting toxicities related to neoadjuvant chemotherapy and radiation therapy, alongside poorer survival outcomes [11]. Go et al. reported that in patients with diffuse large B-cell lymphoma, chemotherapy-induced AEs are increased in patients with low PNI [24]. Chen et al. reported that low PNI was associated with worse prognosis and AEs such as anemia, leukopenia, and myelosuppression in breast cancer patients treated with neoadjuvant chemotherapy [25]. These reports are consistent with our present study and provide a rationale for expanding the predictive effect of PNI on AEs further into the use of PARPi.

Some studies have also investigated the association between mGPS and treatment-related AEs. Draeger et al. reported that mGPS was useful in predicting prognosis and AEs in penile cancer patients undergoing chemotherapy [22]. Freitas et al. also reported that mGPS was useful in predicting prognosis and irAEs in non-small-cell lung cancer patients treated using immune checkpoint inhibitors [26]. These reports also support our finding that a higher mGPS is associated with a higher risk of treatment discontinuation due to treatment-related AEs.

Higher CONUT scores indicate poor nutritional status. Kono et al. reported that a CONUT score ≥5 was predictive of severe AEs in patients with head and neck cancer treated by radiotherapy [23]. In our study, none of the patients had a CONUT score ≥5. The discrepancy could be due to the fact that our study population consisted entirely of patients receiving maintenance therapy, who might be in relatively better health compared to those undergoing more intensive treatments like radiotherapy. Another possible reason the CONUT score was not a useful predictor in our study could be its inclusion of total cholesterol. While PNI and mGPS have been reported in several reports to be useful in predicting treatment-related AEs in cancer patients [11,22,24,25,26], to the best of our knowledge, only one study has evaluated the usefulness of CONUT scores in predicting these events [23]. The key difference between CONUT and the other two indicators lies in the inclusion of cholesterol. Based on our findings, cholesterol may not be useful in terms of predicting treatment-related AEs. In addition, it has been reported that higher cholesterol is associated with a higher risk of cancer proliferation, metastasis and resistance to anti-cancer agents in patients with ovarian cancer [27]. Although the CONUT score indicates that higher cholesterol is associated with better nutritional status, this does not necessarily translate into better outcomes in patients with ovarian cancer. In this context, the inclusion of cholesterol as a marker may be less sensitive to the subtle nutritional changes that affect long-term treatment tolerability, reducing the overall predictive power of the CONUT score for AEs during maintenance therapy. Consequently, other markers like PNI and mGPS, which focus more directly on immune function and systemic inflammation, may provide more accurate predictions in this setting.

Given the influence of nutritional status on treatment discontinuation, several interventions may be considered when PNI is low or mGPS is high at the time of PARPi initiation. The first is to follow high-risk cases more frequently than usual to detect adverse events as soon as possible. Early detection of adverse events and symptomatic treatment is important to ensure continuation of maintenance therapy. The second is dose adjustment. Francis et al. reported no difference in prognosis when PARPi was reduced mid-treatment due to adverse events [28]. This raises the possibility that starting with a reduced initial dose may not change the prognosis for patients assessed to be at high risk of discontinuation due to adverse events at the start of maintenance treatment. Prospective studies are needed to investigate this hypothesis.

Furthermore, our findings suggest that the treatment discontinuation due to AEs with PARPi is influenced by nutritional status at the start of maintenance therapy. Therefore, nutritional intervention prior to the start of maintenance therapy may improve compliance with these drugs. Nutritional interventions have been shown to improve clinical outcomes and are recommended in guidelines for vulnerable older cancer patients [29,30]. In addition, relative intensity studies have suggested that appropriate interventions and adjustment of treatment intensity may contribute to maintaining patient QOL when AEs occur during PARPi administration [28,31]. If changes in treatment intensity do not affect survival, maintaining QOL and improving treatment tolerance are important considerations, which may require assessment of nutritional status and subsequent nutritional interventions.

This study showed several limitations. First, selection biases may have been present, due to the retrospective design of the study. The fact that the decision to discontinue treatment was made by individual attending physicians at each institution may have also contributed to selection bias. Second, daily dietary status and supplement use are important confounding factors in assessing the usefulness of nutritional assessment indicators, but this information was not available. Third, although consecutive patients were enrolled, the study involved a relatively small cohort. The usefulness of nutritional assessment indicators in predicting treatment discontinuation due to AEs with PARPi requires further investigation and validation in prospective studies.

## 5. Conclusions

In conclusion, as PARPi become increasingly integral to maintenance therapy for ovarian cancer, identifying patients at higher risk for AEs is crucial. Our study is the first to suggest that PNI and mGPS can help predict the risk of PARPi maintenance therapy discontinuation due to AEs prior to the treatment. This insight opens avenues for more personalized treatment plans, potentially improving patient outcomes. Further research to validate our findings could refine patient selection for PARPi maintenance therapy, especially for those with low PNI, enhancing both safety and effectiveness.

## Figures and Tables

**Table 1:** Clinical features and treatment of the study population.

	n = 71
Age, median [IQR]	63 [56–69.5]
Age, n (%)	
<65	41 (57.7)
≥65	30 (42.3)
BMI (kg/m^2^), median [IQR]	21.91 [19.6–24.6]
BMI (kg/m^2^), n (%)	
<18.5	12 (16.9)
≥18.5	59 (83.1)
FIGO stage, n (%)	
I or II	5 (7.0)
III or IV	66 (93.0)
Primary tumor location, n (%)	
Ovary	51 (71.8)
Fallopian tube	14 (19.7)
Peritoneum	6 (8.5)
Previous lines of platinum therapy, n (%)	
<3	52 (73.2)
≥3	19 (26.8)
PARP inhibitor ^a^, n (%)	
Olaparib	53 (74.6)
Niraparib	18 (25.4)
Initial dose ^b^, n (%)	
Standard	66 (93.0)
Reduction	5 (7.0)

All patients received PARPi (Olaparib or Niraparib) maintenance therapy for ovarian cancer from January 2018 to December 2023. ^a^ The decision to use either olaparib or niraparib was made by the physician. ^b^ Physicians reduced the initial dose of PARPi due to bone marrow suppression from preceding treatment or age. BMI, body mass index; FIGO, International Federation of Gynecology and Obstetrics; PARP inhibitor, poly (ADP-ribose) polymerase inhibitor.

**Table 2:** Summary of adverse events.

Adverse Events	Any Grade (n, %)	Grade 1	Grade 2	Grade 3	Grade 4	Grade 5
Total	59 (83.1)	36 (50.7)	19 (26.8)	22 (31.0)	4 (5.6)	1 (1.4)
Anemia	16 (22.5)	1 (1.4)	4 (5.6)	11 (15.5)	1 (1.4)	-
Neutropenia	11 (15.5)	-	1 (1.4)	9 (12.7)	1 (1.4)	-
Thrombocytopenia	14 (19.7)	5 (7.0)	3 (4.2)	5 (7.0)	1 (1.4)	-
Creatinine increased	8 (11.3)	4 (5.6)	4 (5.6)	-	-	-
Elevated ALT	2 (2.8)		1 (1.4)		1 (1.4)	
Elevated AST	2 (2.8)		1 (1.4)		1 (1.4)	
Hypertension	2 (2.8)	-	-	2 (2.8)	-	-
Pruritus	1 (1.4)	1 (1.4)	-	-	-	-
Rash maculo-papular	1 (1.4)	1 (1.4)	-	-	-	-
Fatigue	17 (23.9)	13 (18.3)	2 (2.8)	2 (2.8)	-	-
Nausea	18 (25.4)	15 (21.1)	3 (4.2)	-	-	-
Diarrhea	1 (1.4)	1 (1.4)	-	-	-	-
Dysgeusia	6 (8.5)	4 (5.6)	2 (2.8)	-	-	-
Dizziness	2 (2.8)	-	2 (2.8)	-	-	-
Peripheral sensory neuropathy	1 (1.4)	1 (1.4)	-	-	-	-
Stomach pain	4 (5.6)	2 (2.8)	1 (1.4)	1 (1.4)	-	-
Arthralgia	2 (2.8)	1 (1.4)	-	1 (1.4)	-	-
Cough	1 (1.4)	1 (1.4)	-	-	-	-
Pneumonitis	1 (1.4)	1 (1.4)	-	-	-	-
Leukemia	1 (1.4)	-	-	-	-	1 (1.4)

Some cases are represented in multiple adverse events at different severities in the same patient. All adverse events were defined and classified by severity using the Common Terminology Criteria for Adverse Events; CTCAE v5.0. ALT, alanine aminotransferase; AST, aspartate aminotransferase.

**Table 3:** Background of patients discontinued due to adverse events with PARP inhibitors.

No	Age	BMI	FIGO Stage	Histology	Previous Lines of Platinum Therapy	Response to Previous Platinum Therapy	PARP Inhibitor	Treatment Duration (Days)	Adverse Events Leading to Discontinuation
1	63	20.9	II	Serous carcinoma	3	PR	Olaparib	78	Stomach pain
2	71	26.1	III	Serous carcinoma	5	PR	Olaparib	889	AML
3	69	24.8	IV	SCC	2	PR	Olaparib	30	Anemia Thrombocytopenia
4	68	21.0	IV	HGSC	2	CR	Olaparib	196	Creatinine increased
5	52	24.3	IV	Serous carcinoma	2	CR	Niraparib	174	Fatigue Arthralgia
6	69	18.1	IV	HGSC	2	PR	Olaparib	21	Creatinine increased
7	61	20.1	III	HGSC	1	CR	Niraparib	69	Elevated ALT and AST
8	63	21.7	I	Mucinous carcinoma	2	CR	Olaparib	108	Anemia
9	66	22.5	III	HGSC	2	CR	Olaparib	175	Anemia Thrombocytopenia
10	63	17.8	III	HGSC	2	CR	Olaparib	140	Anemia Neutropenia
11	51	17.2	IV	Clear cell carcinoma	3	PR	Niraparib	77	Thrombocytopenia
12	74	26.7	IV	LGSC	2	PR	Olaparib	126	Fatigue
13	69	22.1	III	Adenocarcinoma	3	PR	Olaparib	183	Anemia
14	53	21.2	III	HGSC	1	CR	Olaparib	169	Pneumonitis
15	70	28.2	III	HGSC	1	PR	Niraparib	35	Anemia
16	55	26.5	III	HGSC	1	PR	Olaparib	15	Fatigue
17	64	19.6	III	Adenocarcinoma	1	PR	Olaparib	113	Anemia
18	79	20.6	IV	HGSC	1	CR	Olaparib	140	Creatinine increased

BMI, body mass index; HGSC, high-grade serous carcinoma; LGSC, low-grade serous carcinoma; CR, complete response; PR, partial response; AML, acute myelogenous leukemia; ALT, alanine aminotransferase; AST, aspartate aminotransferase.

**Table 4:** Cox regression analysis of factors related to time to treatment discontinuation (all patients, n = 71).

Factors	Univariate	*p*-Value	Multivariate	*p*-Value
	HR [95%CI]		HR [95%CI]	
Age ≥ 65	1.307 [0.517–3.301]	0.571		
BMI < 18.5	0.959 [0.275–3.337]	0.947		
FIGO stage III or IV	0.436 [0.098–1.935]	0.275		
Previous lines of platinum therapy > 2	0.809 [0.264–2.478]	0.710		
Initial standard dose	0.043 [0.000–57.142]	0.391		
PNI ≤ 48.44	3.867 [1.272–11.761]	0.017	4.232 [1.358–13.191]	0.013
CONUT ≥ 2	2.153 [0.511–9.069]	0.296		
mGPS ≥1	3.554 [1.301–9.711]	0.013	3.678 [1.309–10.333]	0.013

BMI, body mass index; PNI, Prognostic Nutritional Index; CONUT, Controlling Nutritional Status; mGPS, modified Glasgow Prognostic Score; HR, hazard risk; CI, confidence interval.

**Table 5:** Cox regression analysis of factors related to time to treatment discontinuation (only olaparib, n = 40).

Factors	Univariate	*p*-Value	Multivariate	*p*-Value
	HR [95%CI]		HR [95%CI]	
Age ≥ 65	1.881 [0.594–5.961]	0.283		
BMI < 18.5	0.335 [0.042–2.639]	0.299		
FIGO stage III or IV	0.410 [0.088–1.920]	0.258		
Previous lines of platinum therapy > 2	0.538 [0.144–2.009]	0.356		
Initial standard dose	0.044 [0.000–858.143]	0.535		
PNI ≤ 48.44	1.805 [0.543–6.003]	0.336		
CONUT ≥ 2	1.290 [0.284–5.861]	0.741		
mGPS ≥1	6.935 [1.669–28.817]	0.008	8.626 [1.946–38.239]	0.005

BMI, body mass index; PNI, Prognostic Nutritional Index; CONUT, Controlling Nutritional Status; mGPS, modified Glasgow Prognostic Score; HR, hazard risk; CI, confidence interval.

## Data Availability

The data presented in this study are available upon reasonable request from the corresponding author.
